# Enhancing critical thinking and emotional coping of nursing students through life skills training

**DOI:** 10.6026/9732063002002091

**Published:** 2024-12-31

**Authors:** Buvaneswari Ramakrishnan, Juliet Sylvia

**Affiliations:** 1 Department of Psychiatric Nursing, KMCH College of Nursing, Coimbatore, Tamilnadu - 641048, India; 2 Department of Community health Nursing, Alagappa College of Nursing, Karaikudi, Tamilnadu - 630003, India

**Keywords:** Critical thinking, emotional coping Life skills training, nursing students

## Abstract

In the dynamic healthcare landscape, nurses must develop life skills beyond clinical expertise to provide effective patient care and ensure professional growth. This study evaluates the effectiveness of a structured life skills training program for first-year B.Sc. Nursing students in Tamil Nadu, India. This true experimental pre-test-post-test design study was conducted in six randomly selected nursing colleges in Trichy district, Tamil Nadu. The sample included 257 first-year B.Sc. Nursing students, with 126 in the experimental group and 131 in the control group. The eight-week Life Skills Training Program consisted of weekly two-hour sessions. The experimental group showed significant improvements in critical thinking (F=64.26, p<0.001), decision making (F=35.02, p<0.001), problem solving (F=77.54, p<0.001) and coping with emotions (F=39.03, p<0.001) compared to the control group. Associations were found between life skills and socio-demographic variables such as the number of friends, hobbies, reason for choosing nursing and interest in the course.

## Background:

In the dynamic landscape of healthcare, the role of nurses extends beyond clinical expertise to encompass a wide range of life skills essential for effective patient care and professional development. Critical thinking, decision making, problem solving and coping with emotions are fundamental competencies that nursing students must develop to navigate the complexities of modern healthcare environments [1]. The transition from secondary education to a professional nursing program presents significant challenges for first-year students. They must rapidly adapt to a rigorous academic curriculum while simultaneously developing the interpersonal and cognitive skills necessary for their future roles as healthcare providers [2]. Recognizing this need, there is growing interest in implementing structured interventions to enhance life skills among nursing students early in their educational journey [3]. Life skills, as defined by the World Health Organization, are "abilities for adaptive and positive behavior that enable individuals to deal effectively with the demands and challenges of everyday life" (WHO) [4]. For nursing students, these skills are particularly crucial as they form the foundation for effective patient care, teamwork and personal resilience in high-stress medical settings. Previous research has demonstrated the positive impact of life skills training on various student populations [5]. For instance, a study by Anderson *et al.* (2008) found that targeted interventions improved problem-solving abilities among undergraduate students Similarly, kaur (2023) reported enhanced critical thinking skills in medical students following a structured training program [6, 7]. However, there is limited research specifically addressing the effectiveness of comprehensive life skills training for first-year nursing students in the Indian context. Therefore, it is of interest to address this gap by evaluating the effectiveness of a structured life skills training program on critical thinking, decision making, problem solving and coping with emotions among first-year B.Sc. Nursing students in Tamil Nadu, India.

## Methodology:

## Research design:

This study employed a true experimental pre-test-post-test design [8, 9] to evaluate the effectiveness of a structured life skills training program on first-year B.Sc. Nursing students.

## Research setting:

The study was conducted in nursing colleges in Tamil Nadu, specifically in the Trichy district. To ensure representation from various areas and prevent contamination among the sample, six nursing colleges were randomly selected from Trichy.

## Sample and sample size:

The target population comprised first-year B.Sc. Nursing students in selected nursing colleges in Trichy district. The accessible population included those students who met the inclusion criteria. Sample size estimation utilized a statistical formula for comparing two independent means, resulting in a minimum required sample size of 108 in each group. The final sample consisted of 126 subjects in the experimental group and 131 subjects in the control group.

## Sampling technique:

A multi-stage cluster sampling technique was employed. First, Trichy district was selected, followed by the random selection of nursing colleges within the district. Six colleges were chosen through simple random sampling, with each nursing college representing one cluster.

## Intervention:

The Life Skills Training Program was conducted over eight weeks. Each of the eight sessions lasted two hours and was held on the same day each week to maintain continuity and ensure maximum participation. The program covered various life skills topics, focusing on critical thinking, decision making and problem solving and coping with emotions.

## Data collection tool:

A self-administered questionnaire was used for data collection, consisting of two parts:

[1] Socio-demographic variables of first-year B.Sc. Nursing students.

[2] Life Skills Assessment Scale (LSAS), a standardized tool developed and validated for assessing life skills.

## Data analysis:

Data were analyzed using both descriptive and inferential statistics. Descriptive statistics were used to summarize the socio-demographic characteristics of the participants. Chi-square tests were employed to compare socio-demographic variables between the experimental and control groups. The Kruskal-Wallis test was used to assess associations between pretest life skills scores and socio-demographic variables. One-way ANOVA with repeated measures was conducted to evaluate changes in life skills scores over time in both groups.

Table 1 shows most participants were 17 years old, with a higher proportion of hostel residents in the experimental group (97.6%) compared to the control group (77.1%). The majority in both groups reported having more than five friends (88.9% experimental, 68.7% control). Reading books emerged as the most common hobby, while Tamil was the predominant medium of instruction (82.4% control, 78.6% experimental). Personal interest was the leading reason for choosing nursing, more prominent in the experimental group (85.7%) than the control group (73.3%). Table 2 shows association between pretest life skills scores and socio-demographic variables. This table examines the associations between socio-demographic variables and pretest scores for critical thinking, decision-making, problem-solving and coping with emotions. Significant associations were found for hobbies with critical thinking (p < 0.001), decision-making (p = 0.006) and problem-solving (p = 0.002). Additionally, reasons for choosing nursing were significantly associated with problem-solving (p = 0.007) and decision-making (p = 0.048).

Table 3 shows Global Life Skills Scores via One-Way ANOVA. This table shows the one-way repeated measures ANOVA results, revealing significant improvements in the experimental group. Critical thinking scores increased significantly (F = 64.26, p < 0.001), as did decision-making (F = 35.02, p < 0.001), problem-solving (F = 77.54, p < 0.001) and coping with emotions (F = 39.03, p < 0.001). The control group showed minimal improvements across these domains.

## Results & Discussion:

This study aimed to assess the effectiveness of a structured life skills training program on critical thinking, decision making, problem solving and coping with emotions among first-year B.Sc. Nursing students in Tamil Nadu, India. The results demonstrate significant improvements in these key life skills following the intervention. Our study revealed interesting associations between certain socio-demographic variables and life skills. Notably, the number of friends, hobbies, reason for choosing nursing and interest in the course were significantly associated with various life skills. This suggests that social connections, personal interests and motivation for pursuing nursing education play crucial roles in the development of life skills. These findings align with previous research by Lin *et al.* (2023), who found that social support and personal interests significantly influenced the development of life skills among undergraduate students [10]. The structured life skills training program showed significant positive effects on all measured life skills. The experimental group demonstrated substantial improvements in critical thinking, decision making and problem solving and coping with emotions compared to the control group. This is evidenced by the highly significant F-values (p<0.001) in the repeated measures ANOVA for the experimental group across all domains. In our study, critical thinking skills showed marked improvement (F=64.26, p<0.001) in the experimental group. This result is consistent with the findings of Jimenez *et al.* (2021), who reported significant enhancement in critical thinking abilities among nursing students following a structured intervention program [11].

The improvement in decision-making skills (F=35.02, p<0.001) observed in our study corroborates the results of Diouf *et al.* (2022) writing a systemic review, who found that targeted training significantly enhanced decision-making capabilities among healthcare professional [12]. Problem-solving skills also showed substantial improvement (F=77.54, p<0.001) in the experimental group. This aligns with the study by Sahebalzamani (2012), which demonstrated the effectiveness of life skills training in enhancing problem-solving abilities among undergraduate students [13]. The significant improvement in coping with emotions (F=39.03, p<0.001) is particularly noteworthy. This finding is in line with the research of Lim Mei Ling (2011), who reported enhanced emotional coping skills among nursing students following a structured intervention program [14]. Interestingly, our study shows improvements even in the control group, albeit to a lesser extent than the experimental group. This could be attributed to the natural progression of skills during the nursing program. The significant improvements across all domains underscore the value of structured life skills training programs in nursing education. These findings suggest that incorporating such programs into the nursing curriculum could substantially enhance students' preparedness for the challenges of their future profession. As highlighted by MacLean *et al.* (2016), developing skills early in nursing education can lead to improved clinical performance and patient outcome [15]. The findings from these studies align closely with our results, highlighting the significant role of emotional intelligence (EI) in enhancing critical thinking and life skills among nursing students. Hasan and Noor (2023) demonstrated a strong correlation (r = 0.60, p < 0.001) between EI and critical thinking, consistent with our observed improvements in critical thinking and decision-making skills [16]. Similarly, Patil and Nadkarni (2022) found that life skill interventions significantly enhanced EI among nurses, supporting our results that structured training programs improve problem-solving and emotional coping skills [17]. Michelangelo's (2015) meta-analysis further corroborates our findings by showing that EI positively impacts critical thinking, leadership and job satisfaction, emphasizing the importance of integrating EI-focused interventions into nursing education for holistic professional development [18]. While our study demonstrates the effectiveness of the intervention, it was limited to a specific geographical area. Future research could explore the generalizability of these findings across different cultural and educational contexts. This study provides strong evidence for the effectiveness of a structured life skills training program in enhancing critical thinking, decision making, problem solving and emotional coping skills among first-year B.Sc. Nursing students. The findings underscore the importance of integrating such programs into nursing curricula to better prepare students for the complexities of modern healthcare environments.

## Figures and Tables

**Table 1:** Distribution & comparison of the socio-demographic variables of first year B.Sc. Nursing students in the experimental and control group (N=257)

**Variables**	**Control [131]**	**Experimental [126]**	**Chi-square value**	**p-value**
**1. Age in years**				
17	61 (46.6%)	68 (54.0%)	1.6	0.449
18	61 (46.6%)	52 (41.3%)		
19	9 (6.9%)	6 (4.8%)		
**2. Place of stay**				
Hostel	101 (77.1%)	123 (97.6%)	24.163	<0.001***
Home	30 (22.9%)	3 (2.4%)		
**3. Number of friends**				
2-Jan	16 (12.2%)	6 (4.8%)	15.608	<0.001***
5-Mar	25 (19.1%)	8 (6.3%)		
>5	90 (68.7%)	112 (88.9%)		
**4. Hobbies**				
Reading books	34 (26.0%)	34 (27.0%)	5.917	0.205
Listening to music	15 (11.5%)	21 (16.7%)		
Dancing	12 (9.2%)	13 (10.3%)		
Drawing	30 (22.9%)	13 (10.3%)		
Playing games	40 (30.5%)	23 (18.3%)		
**5. Medium of instruction in higher secondary education**				
Tamil	108 (82.4%)	99 (78.6%)	0.614	0.433
English	23 (17.6%)	27 (21.4%)		
**6. Reason for choosing nursing**				
Own interest	96 (73.3%)	108 (85.7%)	6.585	0.037*
Parents force	25 (19.1%)	11 (8.7%)		
Other reasons	10 (7.6%)	7 (5.6%)		
**7. Interest in the course**				
Interested	121 (92.4%)	119 (94.4%)	0.449	0.503
Not interested	10 (7.6%)	7 (5.6%)		
*Statistically significant
*** p<0.001, * p<0.05

**Table 2:** Association between Pretest Life Skills Scores and Socio-demographic Variables of First Year B.Sc. Nursing Students

**Socio-demographic Variable**	**Critical Thinking [Kw Test, p-value]**	**Decision Making [Kw Test, p-value]**	**Problem Solving [Kw Test, p-value]**	**Coping with Emotions [Kw Test, p-value]**	**Coping with Stress [Kw Test, p-value]**
Age	0.895, 0.639	0.102, 0.950	1.659, 0.436	0.242, 0.886	2.536, 0.281
Place of Stay	2.031, 0.362	1.231, 0.540	3.781, 0.152	0.921, 0.630	1.451, 0.484
Number of Friends	1.678, 0.432	6.497, 0.039*	0.472, 0.790	0.750, 0.687	2.729, 0.256
Hobbies	17.85, <0.001*	14.374, 0.006*	16.936, 0.002*	1.890, 0.756	9.232, 0.056
Medium of Instruction	23.41, 0.51	36.21, 0.338	30.03, 0.065	33.89, 0.772	25.28, 1.030
Reason for Choosing Nursing	0.460, 0.794	6.073, 0.048*	10.027, 0.007*	2.281, 0.320	10.352, 0.006*
Interest in the Course	5.319, 0.021*	8.967, 0.003*	5.803, 0.016*	1.375, 0.241	7.854, 0.005*
Domicile	0.297, 0.586	0.651, 0.420	4.930, 0.026*	1.314, 0.252	0.236, 0.627
*-significant at p<0.05
***-significant at p<0.001

**Table 3:** One way ANOVA repeated measures test for Global Life Skills Scores of the experimental and control group (N=257)

**Measure**	**Group**	**F-value**	**p-value**
Critical Thinking	Experimental	64.26	<0.001***
	Control	23.69	<0.001***
Decision Making	Experimental	35.02	<0.001***
	Control	6.42	0.0138
Problem Solving	Experimental	77.54	<0.001***
	Control	39.32	<0.001***
Coping with emotions	Experimental	39.03	<0.001***
	Control	3.292	<0.001***
*** p<0.001
